# Selection of key recommendations for quality indicators describing good quality outbreak response

**DOI:** 10.1186/s12879-015-0896-x

**Published:** 2015-03-31

**Authors:** Evelien Belfroid, Jeannine LA Hautvast, Mirrian Hilbink, Aura Timen, Marlies EJL Hulscher

**Affiliations:** Radboud university medical center, Radboud Institute for Health Sciences, Academic Collaborative Centre AMPHI, Department of Primary and Community Care, PO box 9101, 6500 HB Nijmegen, The Netherlands; Radboud University Medical Center, Radboud Institute for Health Sciences, IQ healthcare, PO box 9101, 6500 HB Nijmegen, The Netherlands; National Coordination Centre for Communicable Disease Control, National Institute for Public Health and the Environment (RIVM), Antonie van Leeuwenhoeklaan 9, 3721 MA Bilthoven, The Netherlands

## Abstract

**Background:**

The performance of recommended control measures is necessary for quick and uniform infectious disease outbreak control. To assess whether these procedures are performed, a valid set of quality indicators (QIs) is required. The goal of this study was to select a set of key recommendations that can be systematically translated into QIs to measure the quality of infectious disease outbreak response from the perspective of disaster emergency responders and infectious disease control professionals.

**Methods:**

Applying the Rand modified Delphi procedure, the following steps were taken to systematically select a set of key recommendations: extraction of recommendations from relevant literature; appraisal of the recommendations in terms of relevance through questionnaires to experts; expert meeting to discuss recommendations; prioritization of recommendations through a second questionnaire; and final expert meeting to approve the selected set. Infectious disease physicians and nurses, policymakers and communication experts participated in the expert group (n = 48).

**Results:**

In total, 54 national and international publications were systematically searched for recommendations, yielding over 200 recommendations. The Rand modified Delphi procedure resulted in a set of 65 key recommendations. The key recommendations were categorized into 10 domains describing the whole response pathway from outbreak recognition to aftercare.

**Conclusion:**

This study provides a set of key recommendations that represents ‘good quality of response to an infectious disease outbreak’. These key recommendations can be systematically translated into QIs. Organizations and professionals involved in outbreak control can use these QIs to monitor the quality of response to infectious disease outbreaks and to assess in which domains improvement is needed.

**Electronic supplementary material:**

The online version of this article (doi:10.1186/s12879-015-0896-x) contains supplementary material, which is available to authorized users.

## Background

Infectious disease outbreaks are a global threat to public health that can have a high economical and societal impact [1,2]. During an outbreak, recommended control measures need to be timely and uniformly performed to curb the spread of pathogens and, ultimately, to reduce the number of persons becoming infected [3].

Numerous documents -from various countries or regions addressing various types of outbreaks- describe such recommended or ‘good quality’ response to infectious disease outbreaks. Unfortunately, publication and dissemination of scripts, guidelines and scientific advice does not guarantee good quality outbreak response [4-6]. Evaluations of recent crises show large variation in the implementation of crisis advice and thus in the quality of outbreak response. A Dutch study examining the response to the Q-fever outbreak showed large regional variance in the implementation of the nationally advised measures [7]. Similarly, evaluation of the control measures during the early stages of the lymphogranuloma venereum (LGV) outbreak in the various EU countries showed great differences between countries with respect to case definitions, laboratory testing and antimicrobial drug treatment [8]. A study examining the causes of differences in the implementation of crisis advice showed that among others, the various categories of professionals involved in outbreak control lacked clearly defined measures to monitor the execution of ‘key actions’ [9].

Quality indicators (QIs) can be used to gain insight into the quality of response and, even more importantly, they can be used to measure the effects of interventions aimed at improving response [10]. In this manner, QIs provide a tool to systematically monitor response quality. Two studies developed QIs for infectious disease outbreak response describing the major domains of outbreak response [11,12]. Both studies lack transparency and reproducibility because they do not provide insight into among others; the selected literature, selected experts and the definition of consensus. It is important that QIs are developed in a systematic and transparent way [10,13,14], which to our knowledge has not been done for infectious disease outbreaks.

The goal of this study was therefore to select a set of key recommendations that can be systematically translated into QIs to measure the quality of infectious disease outbreak response from the perspective of disaster emergency responders and infectious disease control professionals (ID-control professionals) using a valid development procedure. We aimed to develop a *generic* set of key recommendations that can be used to measure the quality of outbreak response, irrespective of the causative pathogen.

## Methods

We used the systematic RAND modified Delphi method to develop and select -in a multistep approach (see Step1 through 5 below)- a set of key recommendations representing good quality infectious disease outbreak response [15]. In this iterative method the individual opinion of experts is aggregated into group consensus. Recommendations for infectious disease outbreak response were extracted from the literature, and presented to a multidisciplinary expert panel. The panel achieved consensus on a set of key recommendations during two questionnaire rounds and two face-to face consensus meetings. Formal ethical approval from a medical ethical committee was not required for this research in the Netherlands since it does not entail subjecting participants to medical treatment or imposing specific rules of conduct on participants. All the experts consented to participate in the study and were aware that their responses would be used for research purposes.

### Step 1 – Literature search and extraction of recommendations

We performed a literature search using the Medline database to review the international literature for information about quality indicators and recommendations for good quality response to an infectious disease outbreak from the year 2007 (search executed 4^th^ week of February 2012). Table 1 shows the search strategy in which we combined the gold standard search strategy of the Cochrane Effective Practice and Organisation of Care Group to identify quality improvement studies and combined these (http://epoc.cochrane.org/) with terms on outbreaks and performance measurement. Two researchers (EB and AT) independently examined title and abstract of the publications to include any publication (for example outbreak reports, evaluations, health services research studies, guidelines) potentially describing recommendations for ID-control professionals and disaster emergency responders. Exclusion criteria were: publications that were not about infectious disease outbreaks (non outbreak setting or no acute outbreak like HIV), publications describing recommendations for a hospital setting, publications that were setting/region or patient specific, and publications that described simulations or mathematical models of outbreaks.

**Table 1:** **Medline search strategy**

The gold standard search strategy of the Cochrane Effective Practice and Organisation of Care group (http://epoc.cochrane.org/)	Communicable diseases OR	Indicator* OR
	communicable disease,	Measurab* OR
	Emerging OR	Performance Measure*
	disease outbreaks OR	OR
	Crisis	Performance
		Assessment* OR
	AND	Quality Assessment* OR
		Quality Measure* OR
	Risk Assessment OR	Manage* OR
	Contact Tracing OR	Recommend* OR
	Disease notification OR	Disease Management OR
	Infection Control OR	Organization OR
	Mandatory Testing OR	Administration OR
	Universal Precautions OR	Scientific AND Advice
	Population Surveillance OR	
	Immunization OR	OR
	Quality Management	
		Quality indicators,
	OR	Healthcare
	Bioterrorism OR	
	Severe Acute Respiratory	
	Syndrome	

Next, we collected grey literature, i.e. Dutch documents on good quality response including national guidance, national outbreak advice, contracts between health care organizations and disaster care plans. We also included national evaluations of recent infectious disease crises such as Q-fever and the 2009 flu pandemic. The inclusion of grey literature was made on the basis of recommendations from national specialists on infectious disease preparedness- and control who were asked to judge appropriateness.

Two researchers (EB and MHi) performed the extraction of recommendations independently on a sample consisting of 25% of all selected sources (literature review and grey literature). The researchers extracted good quality response recommendations from the selected literature. Discrepancies between the two researchers were discussed until consensus was reached. After reaching consensus on this 25% sample, one researcher continued to extract recommendations from the remaining selected literature (EB). The two researchers examined the total set of recommendations to remove identical recommendations.

All recommendations were discussed with the main researchers involved in this study (MHu, JH, AT, EB) in two meetings. In these meetings we selected in consensus and while applying the inclusion criteria, existing generic recommendations or aggregated pathogen and disease specific recommendations, which were subsequently presented to the participants in the expert panels during the next stages.

### Step 2 – First questionnaire round

The consensus procedure took place between September 2012 and May 2013. We approached all 25 public health regions from the Netherlands by e-mail and invited public health infectious disease experts from their region to participate in the expert group. Our expert panels consisted of 48 Dutch experts in public health (28 ID-control professionals and 20 disaster emergency responders) who all had experience in the preparedness and/or control of an infectious disease outbreak. All regions were represented.

Two digital Limesurvey (a digital open source survey application) questionnaires were composed, one for the ID-control professionals and one for the disaster emergency responders. In this process, recommendations were assigned to the responsible organization in the Netherlands: logistical support recommendations were presented to disaster emergency responders and infectious disease control recommendations were presented to ID-control professionals. In the Netherlands, the disaster emergency responder tasks lay with charge of logistical support of outbreak control while the coordination of outbreak measures is a responsibility of the ID-control experts. This, of course, can be different in other countries.

Both expert groups followed a parallel, methodologically identical path: each expert group assessed the recommendations regarding *their* expertise on relevance. Relevance was graded by the experts in response to the following question; “To what extent do you consider this recommendation as a relevant element for measuring the quality of infectious disease outbreak response?” on a 9-point Likert scale (1 = totally disagree, 9 = totally agree). Experts could comment on recommendations and could add recommendations. Recommendations were accepted or further processed based on the RAND/University of California at Los Angeles agreement criteria [15]. Relevance scores were calculated for each item. If the recommendation had a median of 8 or 9 and >70% of the experts scored in the top tertile, then the recommendation was marked as “accepted”. If the recommendation had a median <8 and <70% scored in the top tertile, then the recommendation was marked as “not accepted” and was excluded. If the recommendation had a median <8 and >70% of the experts scored in the top tertile or the median was 8 or 9 and <70% of the experts scored in the top tertile, then the recommendation was marked as “to be discussed”.

In the second part of the questionnaire we checked whether we assigned recommendations to the correct responsible organization (disaster emergency responders or ID-control experts). If more than 33% of the responders questioned the attribution of responsibility for the action to a certain party, then the recommendation was presented to the responsible organization in the second questionnaire round.

### Step 3 – Consensus meeting

Both expert groups were invited for a separate face-to-face consensus meeting in October 2012. The results of the analysis of the questionnaires were sent to the experts in advance of the consensus meeting. During the meeting, the expert panels could comment on recommendations with the label “to be discussed”. As a result, the discussed recommendations were found to be not relevant or were modified and found relevant.

### Step 4 – Second questionnaire round

In step 4, two responsible organization specific questionnaires were composed of all recommendations. To categorize the recommendations, we combined two frameworks which incorporate the main domains of emergency response [16,17]. The recommendations were categorized into 35 categories (25 for ID-control experts and 10 for disaster emergency responders) which described each step in the process of infectious disease outbreak response. These 35 categories or subdomains represented 10 main domains: “Scale of the outbreak and epidemiology”, “Control measures”, “Diagnostics”, “Logistic support”, “Aftercare and conclusion”, “Communication”, “Logistics”, “Upscaling”, “Coordination of the chain of outbreak control” and “Continuity of care”.

Corrections on the assigned responsible organization based on the first questionnaire were processed in this second questionnaire. Doubles were removed if there was too much resemblance of the recommendations within one category. If a number of recommendations represented the same recommendation, but for different patient groups (for example, confirmed case, contact of cases) the recommendations were merged. We asked both expert groups to prioritize the most important recommendation per category, and we calculated the percentage of experts that selected a recommendation. If more than 15 percent of the experts selected a recommendation, the recommendation was considered prioritized. Each group only prioritized recommendations regarding their own expertise.

### Step 5 – Second consensus meeting

In a final, combined face-to-face expert meeting the experts were presented the combined selected sets of quality indicators from step 4. Experts were asked to judge the completeness of the selected recommendations. As a result, recommendations were textually modified, merged or deleted.

## Results

### Step 1 – Literature search and selection of recommendations

**Figure 1:**
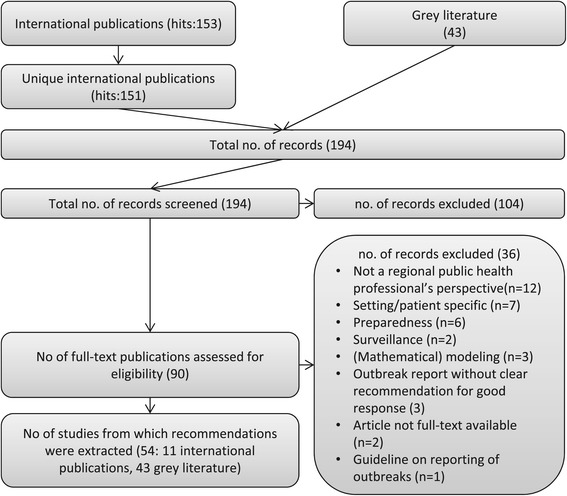
**Flowchart of included and excluded publications.**

The Medline search for international literature regarding quality indicators and recommendations for good quality response to an infectious disease outbreak resulted in 151 unique publications (two identical publications were removed), see Figure 1. Based on title and abstract, 106 of these international publications were excluded while applying exclusion criteria. The remaining 47 publications [18-64] were read full text. From 11 [24,26,27,33,37,38,48,51,58,60,64] out of these 47 articles, we extracted 35 recommendations. The national specialists on infectious disease preparedness- and control plans provided 43 grey literature documents (see Additional file 1), from which we extracted 1057 recommendations. In total, we extracted 1092 recommendations from both the international and the grey literature.

**Figure 2:**
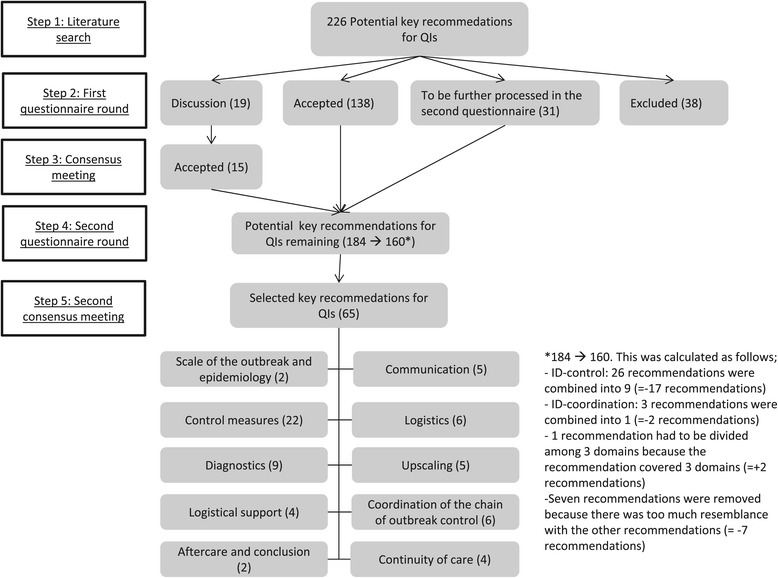
**Selection of key recommendations for quality indicators by the RAND modified Delphi procedure.**

After removal of doubles and of recommendations that were not executed by ID-control professionals and disaster emergency responders, 656 recommendations remained of which 35 were extracted from the international literature. These recommendations were discussed with all researchers involved in this study to make the recommendations generic so that they apply to all outbreaks irrespective of the nature of the causative pathogen and removed doubles while applying the inclusion criteria. This resulted in 226 recommendations (155 for ID-control professionals and 71 for disaster emergency responders). Figure 2 displays the results from the consensus procedure.

### Step 2 – First questionnaire round

Forty-four experts returned the first questionnaire (response rate 92%) containing the 226 recommendations resulting from step 1. After applying the RAND/University of California at Los Angeles agreement criteria, the first consensus round resulted in 138 “accepted” recommendations. Thirty-eight recommendations were found “not accepted”, and therefore excluded. Thirty-one recommendations were marked as “to be further processed in the second questionnaire”.

Both the “accepted” recommendations and the “to be further processed in the second questionnaire” recommendations (N = 169) were subsequently included in the second questionnaire (discussed further in step 4). Nineteen recommendations were marked “ to be discussed ”, meaning that the experts did not reach consensus on the relevance or non-relevance of the items. These recommendations were further assessed in step 3.

### Step 3 – Consensus meeting

In the third step, the expert panels could comment on recommendations with the label “to be discussed” in a consensus meeting. Fourteen ID-control professionals (14 out of 28) and 16 disaster emergency responders (16 out of 20) attended the consensus meeting. The ID-control professionals discussed nine recommendations, of which one was found not relevant, seven were found relevant after textual modification and one was found relevant without modification.

The disaster emergency responders discussed ten recommendations of which two were found not relevant. Two recommendations were combined into one and as a result, seven recommendations were found relevant after textual modification.

In total fifteen recommendations were accepted for further assessment.

### Step 4 - Second questionnaire round

Recommendations were combined into one if the recommendations represented the same action but a different target group (for instance management of suspected case, case, confirmed case). This resulted in a reduction from 184 to 160 recommendations. The prioritization questionnaire consisted of 160 recommendations, of which 31 had to be appraised by both the disaster emergency responders and the ID-control professionals as in practice both could be made responsible for performing the recommended activity.

Forty experts returned the second questionnaire (response rate 88.9%). As a result, the ID-control professionals selected 55 recommendations, the disaster emergency responders 21. In some cases both types of experts prioritized the same recommendation. Deleting those resulted in 69 unique recommendations from both expert groups.

### Step 5 – Second consensus meeting

In the final consensus meeting, both expert panels were merged. Seventeen experts (17 out of 48) attended the consensus meeting. The group discussed the 69 recommendations resulting from step 4. Three recommendations were deleted, 1 newly added and 4 combined into 2. This resulted in 65 recommendations divided among 10 main domains (Table 2): Scale of the outbreak and epidemiology (n = 2), Control measures (n = 22), Diagnostics (n = 9), Logistical support (n = 4), Aftercare and conclusion (n = 2), Communication (n = 5), Logistics (n = 6), Upscaling (n = 5), Coordination of the chain of outbreak control (n = 6) Continuity of care (n = 4).

**Table 2:** Selected key recommendations*

**Domain**	**Quality indicator**
Scale of the outbreak and epidemiology	The [RESPONSIBLE ORGANISATION] collects data about all cases and contacts of cases for inclusion in the case register of the [RESPONSIBLE ORGANISATION] if the nature and phase of the outbreak are such that the data are relevant to outbreak control
Scale of the outbreak and epidemiology	The [RESPONSIBLE ORGANISATION] reports the data about the index case, the epidemiological situation (number and nature of the contacts, possible source, etc.) to the National Authority in charge of *infectious disease control* for national surveillance and coordination
Control measures	The [RESPONSIBLE ORGANISATION] is in charge of supplying prophylaxis for the designated groups
Control measures	The [RESPONSIBLE ORGANISATION] informs the supervisory pharmacy about delivery of the prophylactic medication by the [RESPONSIBLE ORGANISATION]. The pharmacist helps set up the medication management system
Control measures	The [RESPONSIBLE ORGANISATION] must register the indications and contraindications of the prophylactic medication by name and national insurance number of the person receiving treatment whenever the [RESPONSIBLE ORGANISATION] provides prophylaxis
Control measures	The [RESPONSIBLE ORGANISATION] informs the supervising pharmacy about any delivery of medicaments by the [RESPONSIBLE ORGANISATION]. The pharmacist helps set up the medication management system
Control measures	The [RESPONSIBLE ORGANISATION] must register the indications and contraindications for the medication for treatment by name and national insurance number of the person receiving treatment when the [RESPONSIBLE ORGANISATION] is in charge of the treatment
Control measures	The [RESPONSIBLE ORGANISATION] vaccinates only the groups with an indication for vaccination in as far as the [RESPONSIBLE ORGANISATION] gives the vaccination
Control measures	The [RESPONSIBLE ORGANISATION] obtains a list of the residents in the outbreak area (the target population) and approaches them whenever this is necessary**
Control measures	The [RESPONSIBLE ORGANISATION] informs the supervisory pharmacy about any vaccine delivery by the [RESPONSIBLE ORGANISATION]. The pharmacist helps set up the medication management system
Control measures	The [RESPONSIBLE ORGANISATION] looks after registering the indications and contraindications for the vaccine by name and national insurance number of the person receiving vaccination whenever the [RESPONSIBLE ORGANISATION] gives the vaccinations
Control measures	The [RESPONSIBLE ORGANISATION] organises quarantine for contacts of cases, people belonging to the risk group, and other healthcare professionals, with adherence to the national guidelines
Control measures	The [RESPONSIBLE ORGANISATION] verifies whether quarantine for contacts of cases, people belonging to the risk group, and other healthcare professionals was carried out with adherence to the national guidelines
Control measures	Within the agreed time span, the [RESPONSIBLE ORGANISATION] must make available a fully equipped quarantine facility that meets the current requirements starting from the moment that the need of such a facility was made known
Control measures	With adherence to the national guidelines, the [RESPONSIBLE ORGANISATION] places cases and people with symptoms in isolation
Control measures	The [RESPONSIBLE ORGANISATION] verifies whether the national guidelines were adhered to when cases and people with symptoms were placed in isolation at home
Control measures	The [RESPONSIBLE ORGANISATION] is in charge of monitoring the medical condition of any suspected case and contacts of cases by telephone and asks the person about his/her symptoms with a view to the causative pathogen. Thus quick action can be taken, should there be any indication that this person has been infected
Control measures	The [RESPONSIBLE ORGANISATION] instructs people exposed to infection to register themselves with the appropriate caretaker if they develop any symptoms (e.g. flu-like symptoms such as fever and conjunctivitis)
Control measures	The [RESPONSIBLE ORGANISATION] ensures that people with symptoms, people belonging to the risk group, contacts of cases, and other healthcare professionals receive instruction about the appropriate measures to prevent infection in consultation with the treating medical practitioner
Control measures	The [RESPONSIBLE ORGANISATION] instructs the general population about the appropriate measures for controlling infection
Control measures	The [RESPONSIBLE ORGANISATION] instructs the carrier how to transport cases in conformance with the national guidelines
Control measures	The [RESPONSIBLE ORGANISATION] instructs its employees to take the prescribed protection measures the way the national guidelines recommend
Control measures	The [RESPONSIBLE ORGANISATION] informs other health care professionals about personal protection measures conforming to the national guidelines
Control measures	The [RESPONSIBLE ORGANISATION] checks whether its employees use the prescribed protection measures the way the national guidelines recommend
Diagnostics	The [RESPONSIBLE ORGANISATION] provides for the correct diagnostics for cases, contacts of cases, people belonging to the risk groups, and people who meet the case definition criteria as determined in the national guidelines
Diagnostics	The [RESPONSIBLE ORGANISATION] makes arrangements with general practitioners (GPs) and the local medical microbiology laboratory for routing the diagnostics
Diagnostics	The [RESPONSIBLE ORGANISATION] takes charge of diagnostics and any additional control measures (conferring with the consultant microbiologist and the GP)
Diagnostics	The [RESPONSIBLE ORGANISATION] confers with the consultant microbiologist of the region in the event of an infectious disease outbreak
Diagnostics	The [RESPONSIBLE ORGANISATION] provides storage of medical materials (e.g. medicines, medical equipment, necessary research materials, and sterile materials) if national instructions to do so are given
Diagnostics	The [RESPONSIBLE ORGANISATION] distributes medical materials if national instructions to do so are given
Diagnostics	The [RESPONSIBLE ORGANISATION] provides triage based on the national criteria whenever there is a medical indication for such triage
Diagnostics	The [RESPONSIBLE ORGANISATION] provides instruction to the medical interagency partners about the triage criteria
Diagnostics	The [RESPONSIBLE ORGANISATION] organises specific areas (high-risk zones) for the transport, assessment, and management of suspected or confirmed cases with adherence to current protocols (this monitoring measure concerns the separation of patient flows)
Logistic support	The [RESPONSIBLE ORGANISATION] has direct access to diagnostic material
Logistic support	The [RESPONSIBLE ORGANISATION] documents where diagnostic materials can be ordered
Logistic support	The [RESPONSIBLE ORGANISATION] continuously has a supply of the necessary materials in stock, in conformance to the criteria, and determines where these materials can be ordered
Logistic support	The [RESPONSIBLE ORGANISATION], the fire department, and the police organise fire safety, security, and traffic flow around mass-meeting sites, in conformance with the mass vaccination plan
Aftercare and conclusion	When the crisis is over, each [RESPONSIBLE ORGANISATION] evaluates the actions that the organisation itself initiated
Aftercare and conclusion	During the outbreak or soon afterwards, the [RESPONSIBLE ORGANISATION] take the initiative to involve all parties concerned in setting up a plan for aftercare
Communication	The [RESPONSIBLE ORGANISATION] provides health education for people with symptoms, people belonging to the risk group, cases, and contacts of cases
Communication	The [RESPONSIBLE ORGANISATION] limits the number of people visiting infected or suspect locations, such as businesses and student homes, as much as possible
Communication	The [RESPONSIBLE ORGANISATION] adheres to the national communication guidelines
Communication	The [RESPONSIBLE ORGANISATION] has a single leader and centrally directed communication during an outbreak
Communication	The [RESPONSIBLE ORGANISATION] posts up-to-date advice, FAQs, and associated hygiene advice on its website (for residents unfamiliar with the local language as well, if relevant) or states where this advice can be found
Logistics	Among themselves, the [RESPONSIBLE ORGANISATIONS] clearly mark out who is responsible for coordinating and carrying out tasks in the context of infectious disease control
Logistics	The [RESPONSIBLE ORGANISATION] records the decision-making process
Logistics	The [RESPONSIBLE ORGANISATION] adheres to the division of tasks between the [RESPONSIBLE ORGANISATION] and the National Authority in charge of *infectious disease control*
Logistics	The [RESPONSIBLE ORGANISATION] makes and updates a prognosis of bottlenecks on the basis of gaps in care, the attack rate, and the specific pandemic groups
Logistics	The [RESPONSIBLE ORGANISATION] works with adherence to the current procedure strategy
Logistics	The [RESPONSIBLE ORGANISATIONS] inform each other in a timely way about signals and events that may lead to an infectious disease crisis
Upscaling	The [RESPONSIBLE ORGANISATION] appropriately upscales if and when an infectious disease crisis occurs
Upscaling	The Director of Public Health directs and coordinates the elements of the clinical pathway
Upscaling	The [RESPONSIBLE ORGANISATION] makes an appraisal based on complexity (safety partners) and capacities (of the supply chain partners) to scale with adherence to the Coordinated Regional Incident Procedure
Upscaling	The [RESPONSIBLE ORGANISATION] composes a regional multidisciplinary team during a potential outbreak situation
Upscaling	The [RESPONSIBLE ORGANISATION] presents an analysis of expected bottlenecks in regional care to the interagency medical and administrative partners of the multidisciplinary consultation team
Coordination of the chain of outbreak control	The physician in charge of infectious disease control handles communications to external colleagues about field-specific aspects, in as far as the [RESPONSIBLE ORGANISATION] is in charge of communication
Coordination of the chain of outbreak control	The [RESPONSIBLE ORGANISATION] agrees with all parties involved on a contact person for people with symptoms, people belonging to the risk group, cases, contacts of cases, and other healthcare providers
Coordination of the chain of outbreak control	If and when the National Outbreak Management Team has convened, the [RESPONSIBLE ORGANISATION] organises a meeting with the regional crisis centre to discuss the results of the Administrative Coordination Consultation that are made known by the National Authority in charge of *infectious disease control*
Coordination of the chain of outbreak control	The [RESPONSIBLE ORGANISATIONS] maintain permanent contact with each other
Coordination of the chain of outbreak control	The [RESPONSIBLE ORGANISATION] ensures from the beginning that communication with all parties involved is clear, complete, and timely
Coordination of the chain of outbreak control	In the early stages of an outbreak, the [RESPONSIBLE ORGANISATION] organises a face-to-face consultation with all the care partners. During this consultation, the expectations of each organisation are expressed and the assignment of roles and tasks is determined
Continuity of care	During an infectious disease crisis, the [RESPONSIBLE ORGANISATION] maintains an overview of the current need for care and care capacity
Continuity of care	The [RESPONSIBLE ORGANISATION] consults with local GPs about the coordination of general practice care (24/7)
Continuity of care	In coordination with the local care provider networks (for example GP networks and ambulance networks), the [RESPONSIBLE ORGANISATION] consults with GPs about how long the upscaled organisation of primary care will still suffice and from what time supplemental packages will be necessary to guarantee continuity
Continuity of care	In coordination with the local care provider networks (for example GP networks and ambulance networks), the [RESPONSIBLE ORGANISATION] alerts all healthcare providers regarding continuation of care and advises them to act in conformance with the agreements (e.g. for home care and nursing-and-care settings)

*This table represents the selected set of key recommendations that can be systematically translated into Quality Indicators (QIs). For each key recommendation, the responsible organization(s) should be determined prior to measuring the set.

**In the Netherlands, the municipality can provide public service organisations with the list of residents and the necessary contact details from the Municipal Personal Records Database.

## Discussion

In this study, we selected a set of 65 key recommendations describing good quality of infectious disease outbreak response, based on scientific and grey literature, while applying the systematic Rand modified Delphi procedure. We selected key recommendations for the broad range of tasks in infectious disease outbreak response, from “control measures” and “diagnostics” to processes such as “coordination of the chain of outbreak control”. These recommendations can be systematically translated into a set of quality indicators. This QI set is a valuable tool to monitor the quality of response to infectious disease outbreak irrespective of the pathogen, and to assess in which domains improvement is needed. To our knowledge, no generic QIs have been systematically developed to measure the quality of infectious disease outbreak response from the perspective of disaster emergency responders and ID-control professionals describing the whole process of infectious disease outbreak response.

Two studies previously developed QIs applicable to some specific components of outbreak response [11,12]. One study focused on organizational aspects like vaccine availability, communication and reporting [11], while the other study focused on the correct and timely detection of the first cases and the initiation of prophylaxis, education and advice to healthcare workers [12]. Although there is some resemblance between the domains selected in these two studies and our study, our key recommendations are more detailed and specific, which is crucial for a valid and reliable assessment of quality but also for selecting targets for improvement.

Our study has some strengths and limitations. The strength is that our panels consisted of 26 and 18 experts with ample experience in outbreak control, ensuring optimal face validity of the key recommendations. Literature describes that 7–15 experts per panel are needed in order to develop a reliable set of indicators, [15]. The experts brought expertise from various fields such as: infectious disease control, policy making, public health administration, contingency planning, public health nursing, and had been involved in regional and national meetings regarding infectious disease control. Diversity of expert panel members leads to consideration of different perspectives and a wider range of alternatives [65].

Preferably, key recommendations are selected combining evidence- and consensus based recommendations, with a strong preference for evidence based [14]. A limitation of our study is that, from the 656 recommendations which served as a basis for the first questionnaire round, only 35 were extracted from the international literature. In addition, the recommendations are merely practice and expert opinion based. Expert opinion is considered to be the lowest degree of evidence [66]. This is in line with Yeager et al., who studied the literature and concluded that “the public health emergency preparedness literature is dominated by nonempirical studies”. They also describe how only 15 percent of scientific literature on infectious disease outbreaks concerns the response to outbreaks [67]. Although this explains the lack of evidence based recommendations in our study, at the same time it stresses the importance of building a knowledge base for ‘good quality’ response to infectious disease outbreaks. Our set of key could be the starting point for such methodologically sound empirical studies. Another limitation of our study is that our results are, for pragmatic reasons, based on judgments by Dutch experts and partly on Dutch grey literature which may reduce transferability to other countries. To reduce this risk as much as possible, we used a large group of experienced experts with various backgrounds. In addition, a considerable part of the Dutch grey literature is based on documents issued by supranational institutions (e.g. the WHO checklist, ECDC or CDC guidelines). We therefore assume that our key recommendations describe essential elements of outbreak response and will have an added value in improving efforts to deliver high quality response to outbreaks. These key recommendations are formulated in such a way that they allow for adaptation to fit the specific organizational structure of different countries. Most countries have their own national guidelines. European countries lacking national guidelines are very likely to use ECDC guidance instead (such as the RAGIDA guidelines or guidance disseminated through Rapid Risk Assessments).

## Conclusion

In conclusion, this study provides systematically selected key recommendations for good quality of infectious disease outbreak response. These recommendations can be systematically translated into QI to measure the quality of infectious disease outbreak response and to assess in which domains improvement is needed. Their consequent application will provide public health organizations with knowledge on where improvement of outbreak response is needed and how to prioritise the efforts to achieve optimal and uniform outbreak control, in order to be prepared for the next crisis.

The most successful indicators for quality improvement are indicators that are measurable, have potential for feasible improvement, are capable of detecting differences in scores and therefore of discriminating between organizations and are applicable to a large part of the population [14]. At this moment, we are performing a study to test the measurability of our set of key recommendations in practice.

## Additional file

Additional file 1:
**Included grey literature.**
