# Supplementary material for: Selection of key recommendations for quality indicators describing good quality outbreak response
Source: BMC Infect Dis. 2015 Mar 31;15:166. doi: 10.1186/s12879-015-0896-x (PMC4397715; doi:10.1186/s12879-015-0896-x)
Supplement: Additional file 1: — Included grey literature. [file 12879_2015_896_MOESM1_ESM.docx]

Additional file 1: Included grey literature

| Types of included grey documents | N (total=43) |
| --- | --- |
| National disease specific outbreak control guidance (for example Influenza and SARS) | 6 |
| National generic outbreak control guidance | 2 |
| Generic disaster emergency responder scripts | 2 |
| Disaster emergency plans | 3 |
| Advices of the national Outbreak Management Team (OMT) from past crises | 15 |
| Quality indicators public health from the Dutch national inspectorate | 1 |
| Certification demands of the Dutch organization for Harmonization of Quality in Healthcare (HKZ) | 2 |
| Model agreements for crises and outbreaks (for example disasters and crises) | 4 |
| WHO Quality indicators | 1 |
| National outbreak evaluations (for example Q-fever and Influenza A H1N1) | 3 |
| Other | 4 |
